# Evaluation of the Protective and Regenerative Properties of Commercially Available Artichoke Leaf Powder Extract on Plasma and Liver Oxidative Stress Parameters

**DOI:** 10.3390/antiox12101846

**Published:** 2023-10-11

**Authors:** Ewa Florek, Marta Szukalska, Katarzyna Markiewicz, Izabela Miechowicz, Justyna Gornowicz-Porowska, Anna Jelińska, Joanna Kasprzyk-Pochopień, Joanna Nawrot, Agnieszka Sobczak, Małgorzata Horoszkiewicz, Wojciech Piekoszewski, Gerard Nowak

**Affiliations:** 1 Laboratory of Environmental Research, Department of Toxicology, Poznan University of Medical Sciences, 60-631 Poznan, Poland; martan@ump.edu.pl (M.S.); katmarkiewicz@wp.pl (K.M.); 2 Department of Computer Science and Statistics, Poznan University of Medical Sciences, 60-806 Poznan, Poland; iza@ump.edu.pl; 3 Department of Practical Cosmetology and Skin Disease Prevention, Poznan University of Medical Sciences, 60-806 Poznan, Poland; justyna.gornowicz-porowska@ump.edu.pl (J.G.-P.); joannac@ump.edu.pl (J.N.); gnowak.gerard@gmail.com (G.N.); 4 Chair and Department of Pharmaceutical Chemistry, Poznan University of Medical Sciences, 60-780 Poznan, Poland; ajelinsk@ump.edu.pl (A.J.); asobczak@ump.edu.pl (A.S.); 5 Laboratory of High-Resolution Mass Spectrometry, Faculty of Chemistry, Jagiellonian University, 30-387 Krakow, Poland; kasprzykj@chemia.uj.edu.pl (J.K.-P.); piekosze@chemia.uj.edu.pl (W.P.); 6 Botaniqa Pharmacy, 60-783 Poznan, Poland; mhoroszkiewicz@botaniqa.com.pl; 7 Department of Analytical Chemistry, Faculty of Chemistry, Jagiellonian University, 30-387 Krakow, Poland

**Keywords:** artichoke, antioxidants, liver, oxidative stress, carbon tetrachloride, animal

## Abstract

Hepatocellular damage by the harmful effects of xenobiotics, which increase the production of free radicals, is a widespread phenomenon. The extract from the leaves of *Cynara scolymus* L. available as an artichoke preparation (natural source) of antioxidants may serve as a potential hepatoprotective factor. This study aimed to evaluate the impact of the protective and regenerative properties of artichoke preparation on the liver in three extract doses: 0.5; 1.0; and 1.5 g/kg bw/day. The evaluation was conducted by measuring the levels of oxidative stress parameters, including glutathione (GSH), glutathione S-transferases (GST), nitric oxide (NO), superoxide dismutase (SOD), catalase (CAT), Trolox equivalent antioxidant capacity (TEAC), thiobarbituric acid reactive substances (TBARS), glutathione peroxidase (GPx), paraoxonase 1 (PON1), SH- group, nitrosylated protein (RSNO), as well as such liver enzymes as alanine aminotransferase (ALT), aspartate aminotransferase (AST), and alkaline phosphatase (ALP) in the plasma and liver homogenate of rats with liver damage induced by CCl_4_ (1 mL/kg bw). Measurements were taken in plasma and liver homogenate. The results have demonstrated that the artichoke preparation, owing to its high antioxidative potential, exhibits protective and regenerative effects on the liver. This is supported by the observation of higher GSH levels in the plasma of rats treated with artichoke extract for two weeks before CCl_4_ exposure. Furthermore, the artichoke extract has shown regenerative properties, as evidenced by lower ALT, AST, and SOD activity in the group treated with artichoke extract after CCl_4_ exposure. These findings suggest that the in vivo administration of artichoke preparation may be beneficial for the protection and regeneration of the liver.

## 1. Introduction 

The imbalance between prooxidants and antioxidants occurs in the body during increased production of reactive oxygen species (ROS) or reduced content of antioxidants. This condition is called oxidative stress. If it persists for an extended period, it can cause serious cell damage [1,2]. Consequently, ROS contributes to the development of many diseases, including cancers, cardiovascular and neurodegenerative disorders, atherosclerosis, diabetes, and liver diseases [1,3,4]. There has been a growing interest in natural sources of antioxidants that support the defense mechanisms of human cells. One such source is artichoke (*Cynara scolymus* L.), a vegetable cultivated for human consumption (the bottom flower) and for medicinal purposes (herbs and leaves), mainly in the Mediterranean region. This plant contains numerous active compounds: phenolic derivatives; flavonoids; phytosterols (β-sitosterol, taraksasterol); sesquiterpene lactones; triterpenes; tannins; carbohydrates (inulin, locks, pectin); enzymes; amino acids; B vitamins; vitamin C; carotenoids; and microelements [5,6,7]. Experiments on animals and human research have shown that artichoke contributes to improving the blood lipid profile, leading to lower cholesterol and triglyceride levels and reducing the ratio of total cholesterol to HDL [8,9,10,11]. Additionally, it stimulates and facilitates the secretion of bile [12,13,14,15]. Studies on cell cultures involving hyperlipidemic patients have demonstrated a positive effect on vascular endothelial function [16,17,18,19]. Due to the presence of fructooligosaccharides, artichoke exhibits prebiotic properties and has a beneficial effect on functional bowel disorders, as well as preventing infections [20,21,22]. The use of artichoke leaf extract reduces glucose levels and possesses anti-inflammatory and anti-cancer properties [11,19,23,24,25]. 

Liver and kidney cells damaged by the harmful effects of xenobiotics, such as coffee, alcohol, cigarettes, drugs, and certain environmental pollutants that increase the production of ROS, are a widespread phenomenon all over the world [26,27,28]. 

This study aimed to evaluate the antioxidant activity (protective and regenerative properties) of a commercially available artichoke leaf extract in the plasma and liver of rats exposed to carbon tetrachloride. 

## 2. Materials and Methods 

### 2.1. Chemicals and Reagents 

All chemicals used for biochemical determinations were of analytical reagent grade (Sigma-Aldrich, Merck, Darmstadt, Germany). All organic solvents used in the HPLC studies were of high-performance liquid chromatographic grade (J.T. Baker, Avantor Performance Materials, Gliwice, Poland). 

### 2.2. Plant Material 

Commercially available artichoke (*Cynara scolymus* L., family Asteraceae) leaf powder extract with a minimum content of caffeoylquinic acids, 2.5%, was manufactured by Martin Bauer Group, Vestenbergsgreuth, Germany (No. 0135112). The extract was obtained through the gentle water extraction process in accordance with applicable regulations and GMP requirements. Artichoke leaf extract was compliant with the European Pharmacopoeia, ensuring safety and effectiveness.

### 2.3. TLC Analysis 

**Figure 1:**
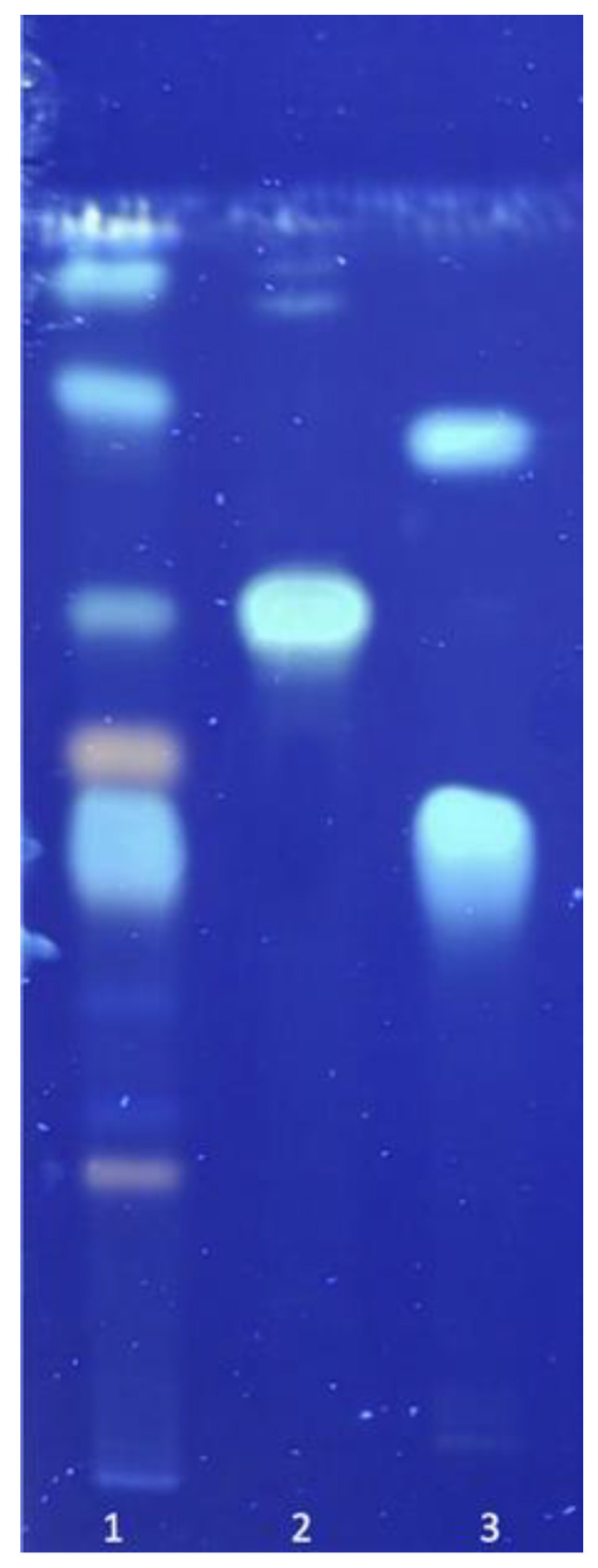
Chromatogram of pharmacologically active compounds from *Cynarae herba*. Mobile phase: Formic acid–water–methyl ethyl ketone–ethyl acetate (1:1:3:5). Description: 1. *Cynarae herba* extract; 2. cynarin; 3. chlorogenic acid.

The preliminary analysis of Cynarae extract was conducted using thin-layer chromatography. Cynarae herba siccus extract was dissolved in ethanol. This study was carried out at room temperature on aluminum-backed silica gel plates (DC Alufolien Kieselgel 60, Merck Art. 5553, Darmstadt, Germany). The identity of cynarin and chlorogenic acid was confirmed by co-chromatography with an authentic sample. For each plate, 15–20 μg of Cynarae extract and each pure compound were applied as a stripe. To detect cynarin and chlorogenic acid, a mobile phase of formic acid–water–methyl ethyl ketone–ethyl acetate (1:1:3:5) was used (Figure 1). After the development and drying of the chromatograms, natural product polyethylene glycol reagent (NEU-reagent) was sprayed to visualize phenol compounds. Cynarae herba extract is characterized by chlorogenic acid; the main compound was seen at Rf ~0.52 and was detected in UV-365 nm as a blue fluorescent zone. Cynarin (Rf ~0.67) was present in low concentrations (Figure 1). 

### 2.4. HPLC Analysis

#### 2.4.1. Chromatographic Conditions

The chlorogenic acid amount in artichoke extract was determined by the HPLC method using an Agilent 1260 Infinity II LC Chromatograph (Agilent Technologies, Bolinem, Germany). This system was equipped with a quaternary pump (model G7111B) and degasser, a vial sampler (model G7129A), a multi-column thermostat (model G7116A) set at 40 °C ± 0.8 °C, and a diode array detector (DAD WR, model G7115A) set at λ = 330 nm. Chromatographic separation was conducted on LiChrosher^®^ 100 RP-18 column (4.6 × 250 mm, 5 μm; Merck KGaA, Darmstadt, Germany) with gradient elution at the 1.2 mL/min flow rate. The mobile phase consisted of a combination of water (phase A) and acetonitrile (phase B), both containing phosphoric acid (0.5 H_3_PO_4_:99.5 solvent; *v*/*v*). The gradient conditions are reported in Table 1. The injection volume was 25 μL.

The above conditions were obtained by modifying the pharmacopeial method of chlorogenic acid determination described in the Artichoke leaf dry extract monograph—European Pharmacopeia (Ph. Eur. 10th Edition). The changes to the gradient program ensured the separation of chlorogenic acid from other components in the extract. The method was validated according to the International Council for Harmonization (ICH) requirements with regard to selectivity, linearity, precision, and accuracy. The stock solutions were prepared by dissolving appropriate amounts of chlorogenic acid in methanol (~0.1 mg/mL). Next, during the validation procedure, stock solutions were further diluted in the mixture of methanol and water (30:70, *v*/*v*) to yield concentrations within the calibration ranges (n = 9) and precision. The precision samples were prepared six times in a concentration (7.4 μg/mL) corresponding to the concentration of the tested analyte (~7 μg/mL). The tested samples (n = 3) were prepared by dissolving the artichoke extract in the mixture of methanol and water (30:70, *v*/*v*) at concentrations of about 1.1 mg of extract per 1 mL of a solvent mixture. The reference solution was prepared by mixing 5.0 mL of chlorogenic acid stock solution and 5.0 mL of methanol and filling up to 20.0 mL with water (~25 μg/mL). The chlorogenic acid content in the extract was calculated according to the pharmacopeia formula (Ph. Eur. 10th Edition).

#### 2.4.2. Validation Results

**Figure 2:**
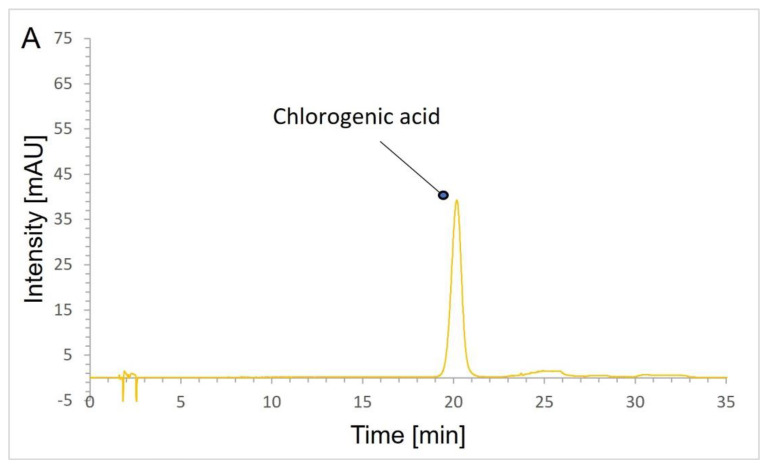

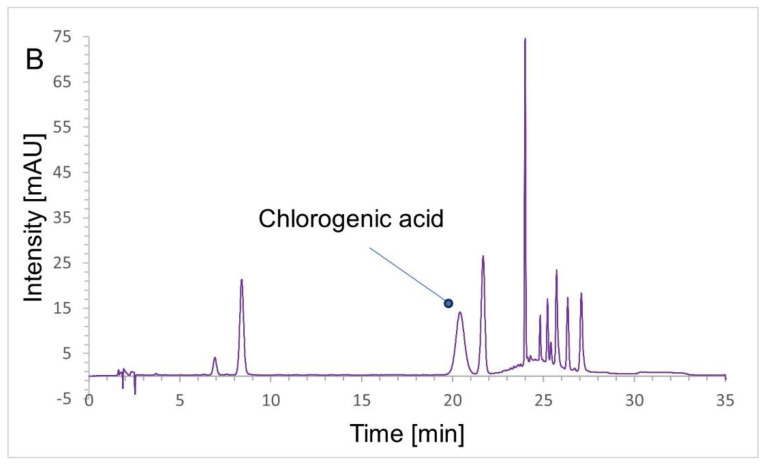
HPLC chromatograms of hydroalcoholic solution of: (**A**)—chlorogenic acid; (**B**)—artichoke extract.

In the definition section of the artichoke leaf dry extract monograph (European Pharmacopeia 10th Edition), the quality of the extract is determined based on the chlorogenic acid content. Therefore, in this research, the above parameter was decided to be checked using a validated HPLC method. The selectivity of the HPLC method was evaluated by verifying the chromatograms of chlorogenic acid, solution of the artichoke extract, and blank samples (mixture of methanol and water). The applied method separated all extract components from chlorogenic acid (Figure 2). The developed method was linear in the range of 2.5–34.3 μg/mL (*y* = a*x*; *r* = 0.9997), and the limits of detection and quantitation were 0.48 μg/mL and 1.45 μg/mL, respectively. The precision of this method expressed by values of relative standard deviation in percentages was satisfactory (RSD% = 1.3% for 7.4 μg/mL). Moreover, the method was accurate, as evidenced by the level of recovery 96.6–99.7%. 

The chlorogenic acid content in the extract was calculated following the methodology indicated in the European Pharmacopeia (Ph. Eur. 10th Edition) and was 0.62 ± 0.004% (n = 3). The obtained value proves that the artichoke extract meets the pharmacopeial requirements in terms of the content of chlorogenic acid.

### 2.5. Ethics Committee Approval 

This study’s design was approved by the Ethics Committee for Animal Experiments Affairs in Poznan, Poland (Approval No. 18/2010). All procedures concerning the handling and use of laboratory animals were performed in accordance with European Union (UE) regulations under Directive 2010/63/EU on the protection of animals used for scientific purposes. Experiments were carried out in accordance with the so-called 3Rs principle (Replacement, Reduction, Refinement) to protect animals. To obtain consistent data, this study was based on the required minimum number of animals and observation time. To improve the rigor and reproducibility of animal research, all data were collected according to ARRIVE 2.0 guidelines. *In vivo* experiments were carried out in the Department of Toxicology of the Poznan University of Medical Sciences in Poznan, Poland. The above-mentioned centre is a unit listed by the Ministry of Education and Science. Contractors had individual permits for planning and performing experiments, as well as the killing of animals. 

### 2.6. Animals and Experimental Treatments 

The experiment was conducted following the guidelines of the Animal Protection Act, which was used for scientific purposes and education (*Journal of Laws* 2015, of 26 February 2015, item 266). These guidelines were based on the EU Directive 2010/63. Experimental animals were obtained from the breeding program of the Department of Toxicology, Poznan University of Medical Sciences. This experiment was carried out on male Wistar rats, which were part of an outbred herd, aged 4 months old, with an average body weight of 350.0 ± 25 g. To minimize the number of animals used in the experiment and follow the 3R principle (Replacement, Reduction, Refinement) aimed at animal protection, only animals of one sex (male) were used. We deliberately limited the number of animals required for testing to the minimum necessary to obtain reliable results. Advanced statistical methods were employed to analyze the data with the minimum number of samples. The procedures and the activities were meticulously planned to minimize the suffering of the animals during the experiments as much as possible. 

Among other things, due to the minimization of the number of animals used for the experiment, rats of one sex were selected. The proposed size of the study groups is the optimal number to maximize the scientific integrity of the generated data while using the minimum number of animals necessary for statistical calculations of test results and drawing appropriate conclusions from the conducted experiment. The animals were properly kept in polypropylene cages (n = 2 rat/cage) with autoclaved pine sawdust litter under controlled environmental conditions (12 h light/ 12 h dark: 6 am–6 pm; temperature: 22 ± 2 °C; air humidity: 50–60%). The animals were allowed to acclimatize for two weeks before the beginning of the experiment with ad libitum access to water and wholesome feed. The animals were fed Labofeed B Plant (“Morawski” Feed Production Plant—the dietary formula was created based on the recommendations of the National Research Council in the field of Nutrient Requirements of Laboratory Animals). 

**Figure 3:**
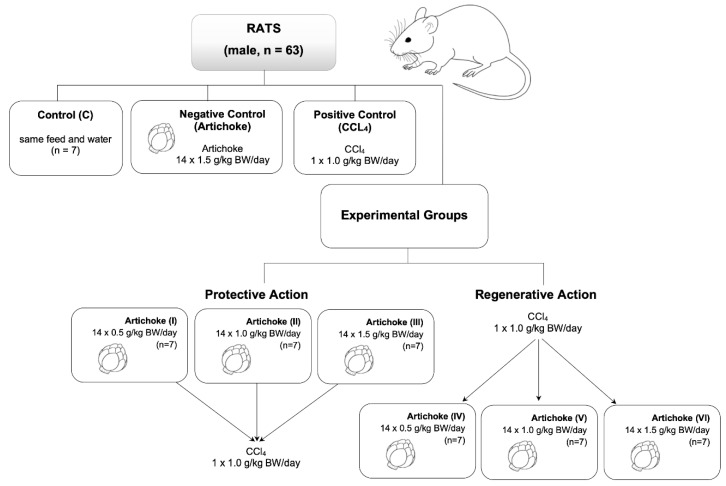
The scheme of the course of the experiment.

After two weeks of acclimatization, the rats were randomly divided into 9 experimental groups (Figure 3), each consisting of 7 male rats. The control group (C) consisted of animals receiving only food and water. Additionally, we had a positive control group comprising seven male rats, which received 14 doses of commercially available artichoke extract each day of 1.5 g/kg. The negative control group (1 × 1 g/kg bw) received carbon tetrachloride. For the study of the protective activity of artichoke aqueous commercial extract, we administrated volumes of 2 mL containing 0.5 g/kg bw (group I), 1.0 g/kg bw (group II), and 1.5 g/kg bw (group III) of commercial artichoke extract for 14 days through a gastric tube. After this treatment, rats received 1 mL/kg bw of CCl_4_. Groups IV, V, and VI (regenerative groups) were animals that initially received 1 mL/kg bw CCl_4_, and during the biweekly experiment, they received an aqueous commercial extract of artichoke leaf. 

For biochemical and toxicological analysis, blood drawn from the hearts and livers of animals on a specific day of the experiment was used. The animals were injected intraperitoneally with a 1:1 (*v*/*v*) combination of ketamine (90 mg/kg; Kepro, The Netherlands) and xylazine (10 mg/kg; Kela, Belgium) to induce anesthesia. After a total of 10 min from the injection, when deep anesthesia was achieved, the animals were euthanized by drawing blood from the heart, and their left lobe liver tissues were removed. Blood was collected in heparinized tubes and centrifuged for 10 min at 3000 rpm/min, after which the plasma was collected and frozen at −80 °C for subsequent determinations. To measure the enzymatic activity and oxidative stress parameters (total protein (TP), ALT, AST, ALP, GST, GSH, NO, SOD, RSNO, TEAC, TBARS, CAT, GPx, PON1 and -SH group), each rat tissue was placed in phosphate-buffered saline (PBS) and stored in the freezer at −80 °C. During the animal experiments, all ethical issues related to working with animals were strictly observed by the guidelines of the Ethics Committee for Animal Experiments Affairs in Poznan, Poland. 

### 2.7. Preparation of Tissue Samples 

An amount of 0.25 g of liver tissue was weighed and placed in 50 mL Falcon tubes. To this, 2 mL of PBS buffer (Sigma-Aldrich, Darmstadt, Germany) diluted 1:9 with saline was added. The tissue was homogenized in a homogenizer at 24,000 rpm and then transferred to 15 mL Falcon tubes. The biological material was centrifuged for 10 min at 4 °C (4200 rpm). The resulting supernatant was transferred to 2 mL Eppendorf tubes and centrifuged again for 10 min at 4 °C (6000 rpm). The resulting supernatant was pipetted into 2 mL Eppendorf tubes. Finally, the biological material prepared in this way was stored in a freezer at −80 °C. 

### 2.8. Biochemical Assays 

Relevant markers of oxidative stress and biochemical parameters were determined in plasma (TP, ALT, AST, ALP, SOD, GSH, NO, -SH groups, RSNO, TEAC, TBARS) and liver homogenate (TP, GST, GSH, CAT, SOD, GPX, RSNO, -SH groups, PON1, TEAC, TBARS) using spectrophotometric methods. 

Total protein (TP) concentration was determined using Lowry’s method—a combination of a biuret test and Folin–Ciocalteu reaction [29]. 

Activities of alanine aminotransferase (ALT), aspartate aminotransferase (AST), and alkaline phosphatase (ALP) were performed using in vitro assays System Roche/Hitachi cobas c. (Roche Diagnostics, Mannheim, Germany, 2012). 

Glutathione S-transferase (GST) enzymatic activity was assessed based on the coupling reaction of thiol groups of L-glutathione with 1-chloro-2,4-dinitrobenzene (CDBN) [30]. 

The reduced glutathione (GSH), the level was determined by the Ellman method (reaction with 5,5-dithio-bis -(2-nitrobenzoic acid, DTNB) [31]. 

By measuring the concentration of stable degradation products, nitrates (V), and nitrates (III) (nitrites) in an aqueous solution, NO concentrations were determined [32]. 

The activity of superoxide dismutase (SOD) was measured by the Randox test—„Ransod–Superoxide dismutase” (Manual Rx Monza—RANSOD—SD 125. RANDOX Laboratories Ltd., Co. Antrim, United Kingdom, 2012). 

The concentration of S-nitrosothiols (RSNO) was determined using the Saville/Gries method [33]. 

The Trolox equivalent antioxidant capacity (TEAC) of substances present in the solutions was measured based on the measurement of stable radical cation reduction capacity (ABTS)•+ [34]. 

Thiobarbituric acid reactive substances (TBARS) measurement was used for monitoring lipid peroxidation [35]. 

The activity of catalase (CAT) was determined based on the reaction of H2O2 degradation. The unit of CAT activity is the enzyme amount that degrades 1 μM H2O2 solution within 1 min, which corresponds to absorbance reduction by 0.036 U/min (volume: 1 mL, optical path length: 1 cm) [30]. 

The activity of glutathione peroxidase (GPx) was determined based on the oxidation reaction of glutathione in the presence of H_2_O_2_ to glutathione disulfide (GSSG), for which GPx is a catalyst. The assumed unit of GPx activity was the enzyme amount that oxidizes 1 μM of GSH (0.5 μM NADPH) within 1 min [30]. 

The arylesterase activity of paraoxonase-1 (PON1) was determined based on the decomposition reaction of phenyl acetate to phenol and acetate anion, which is catalyzed by PON1 [36]. 

The sulfhydryl groups (-SH) were determined by reaction with Ellman’s reagent [30]. 

### 2.9. Statistical Analysis 

The results were subject to statistical analysis using Statistica 10 (StatSoft, Krakow, Poland). To characterize the measured parameters, the arithmetic mean and the standard deviation were used in each group. To analyze the differences between groups, normality tests and analysis of variance (ANOVA) were used. In the case of variables not following a normal distribution and lacking equal variance, the Kruskal–Wallis’s test was applied. 

## 3. Results 

Changes in enzyme activities and concentration of the oxidative stress markers in plasma were assessed in nine groups of seven laboratory animals (male rats) (Figure 3). The following groups were investigated: the control group (receiving water); the positive control group (receiving the highest concentration of artichoke preparation—1.5 g/kg bw—used in this study); and the negative control group containing the dose of carbon tetrachloride (1 × 1 mL/kg bw) used in the tests. Groups I–III assessed the protective effect of artichoke, while groups IV–VI assessed the regenerative effect of the tested preparation. 

**Figure 4:**
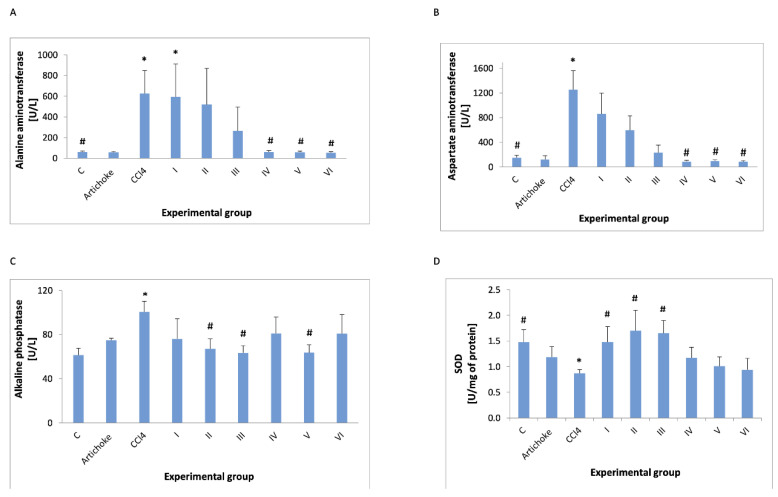
ALT (**A**), AST (**B**), ALP (**C**), and SOD (**D**) activities in plasma in all experimental groups; * statistically significant difference in the studied groups versus the control group; ^#^ statistically significant difference in the studied groups versus group after administration of carbon tetrachloride.

**Figure 5:**
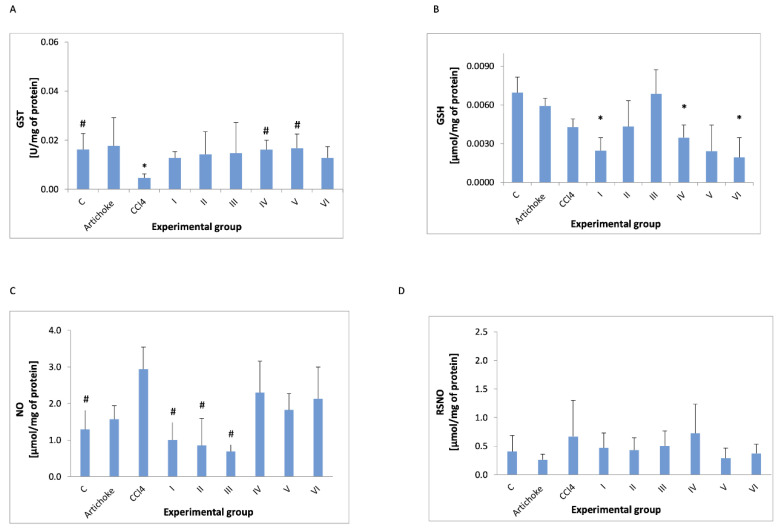
GST (**A**) activity and concentration in plasma of GSH (**B**), NO (**C**), and RSNO (**D**) in all experimental groups; * statistically significant difference in the studied groups versus control group; ^#^ statistically significant difference in the studied groups versus group after administration of carbon tetrachloride.

The analysis of the enzyme activities in plasma (ALT, AST, ALP, GST, and SOD) showed the protective effect of the artichoke preparation (Figure 4 and Figure 5). 

The analysis of enzyme activities in plasma (ALT, AST, ALP, GST, and SOD) showed the regenerative effect of the artichoke preparation (Figure 4 and Figure 5). ALT (EC 2.6.1.2) and AST (EC 2.6.1.1) activities increased after the administration of CCl_4_, but they returned to the values observed in the control group after the administration of the artichoke preparation during the regeneration phase. ALP (EC 3.1.3.1) also significantly increased after the administration of CCl_4_, and the administration of the artichoke preparation caused a statistically significant decrease at a dose of 1 g/kg bw. SOD (EC 1.15.1.1) decreased after the administration of CCl_4_, and after the administration of artichoke extract, it increased, but the increase was not statistically significant. GSH—administration of an artichoke preparation after prior exposure to CCl_4_ did not return to GSH levels to those seen in controls. 

SOD decreased after the administration of CCl_4_. However, the earlier administration of artichoke preparation significantly increased the activity of SOD, bringing it to values similar to those observed in the control and positive control groups (Figure 4). 

**Figure 6:**
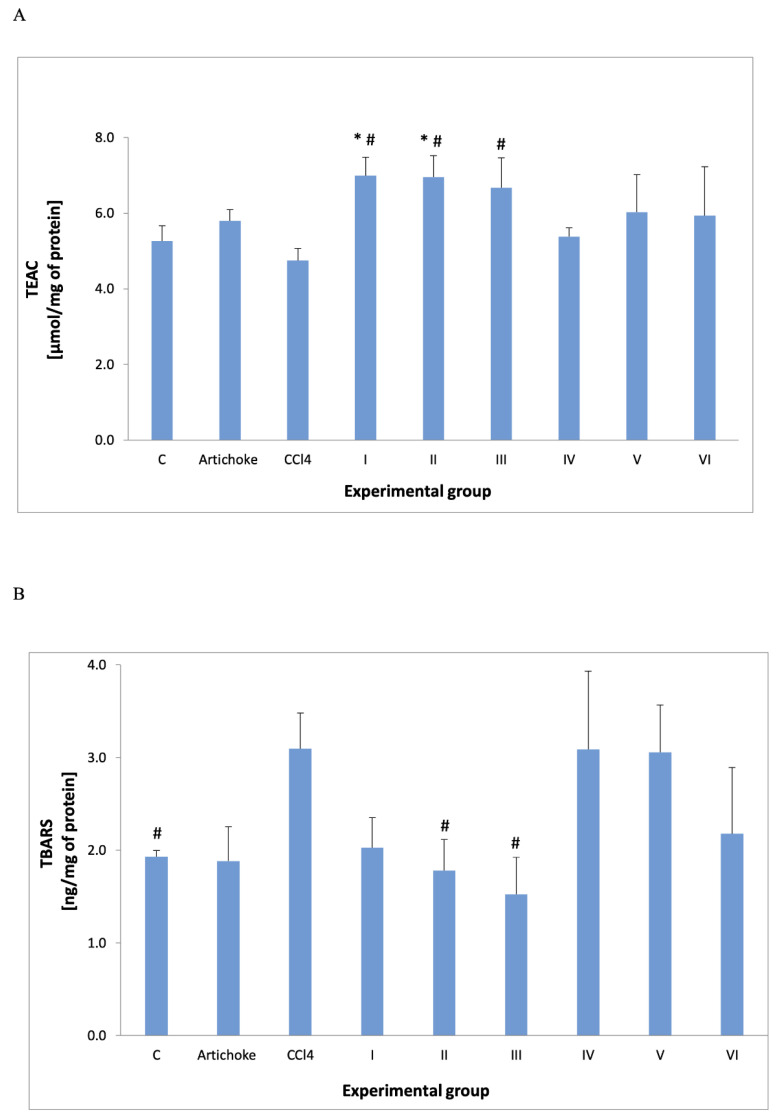
TEAC (**A**) and TBARS (**B**) result in plasma. * statistically significant difference between the studied groups versus control group; ^#^ statistically significant difference in the studied groups versus group after administration of carbon tetrachloride.

In addition to enzyme activity, key parameters of oxidative stress were also examined (Figure 5 and Figure 6). The administration of CCl_4_ lowered the GSH concentration, but this change was not statistically significant. However, the administration of 1.0 and 1.5 g/kg bw of the artichoke preparation resulted in the return of the GSH level to that of the control group, and this change was also not statistically significant. GST activity is lowered by CCl_4_ and is close to the values observed in artichoke after protective administration of the artichoke preparation at the lowest dose used in the study—0.5 g/kg. After administration of CCl_4_, a statistically insignificant increase in NO level was observed in the control group, while all doses of the artichoke preparation caused the NO level to return to that observed in the control group. Neither the administration of CCl_4_ nor the subsequent administration of the artichoke preparations changed the level of nitrosylated protein. 

The administration of artichoke after the CCl_4_ administration did not change the NO levels to that seen in controls. No statistically significant differences were found for RSNO. Variable changes in the level of nitrosylated protein were observed as a result of the tested artichoke preparation. 

Trolox equivalent antioxidant capacity increased in groups I and II compared to the control group and in groups I to III compared to the group after administration of carbon tetrachloride. The thiobarbituric acid reactive substances were significantly increased after the administration of CCl_4_, but the administration of 1.0 and 1.5 g/kg bw of the artichoke preparation lowered this result significantly compared to the group to which carbon tetrachloride was administered. 

**Figure 7:**
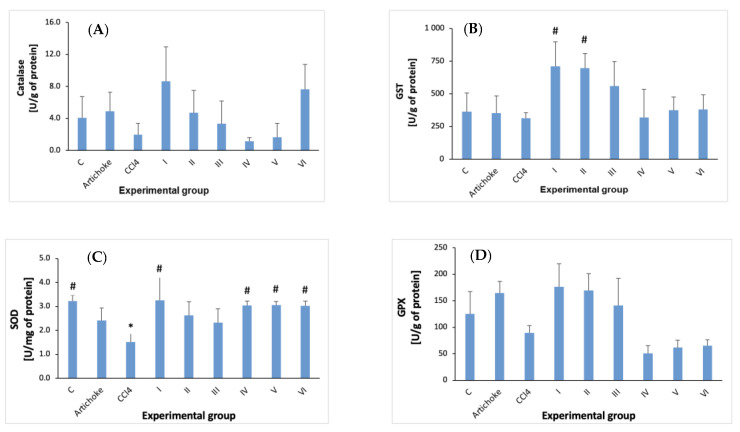
Catalase (**A**), GST (**B**), SOD (**C**), and GPX (**D**) activity in the liver homogenate in all experimental groups; * statistically significant difference in the studied groups versus control group; ^#^ statistically significant difference in the studied groups versus group after administration of carbon tetrachloride.

**Figure 8:**
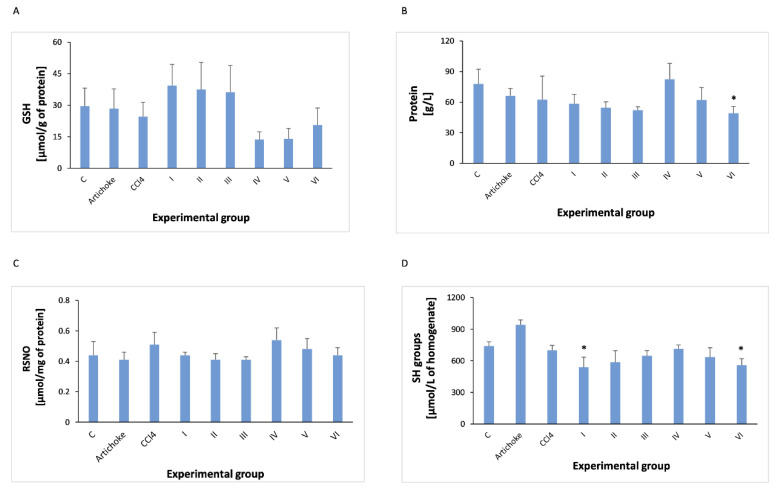
Concentrations of GSH (**A**), TP (**B**), RSNO (**C**), and -SH groups (**D**) in the liver homogenate of all experimental groups; * statistically significant difference in the studied groups versus control groupin the studied groups versus group after administration of carbon tetrachloride.

**Figure 9:**
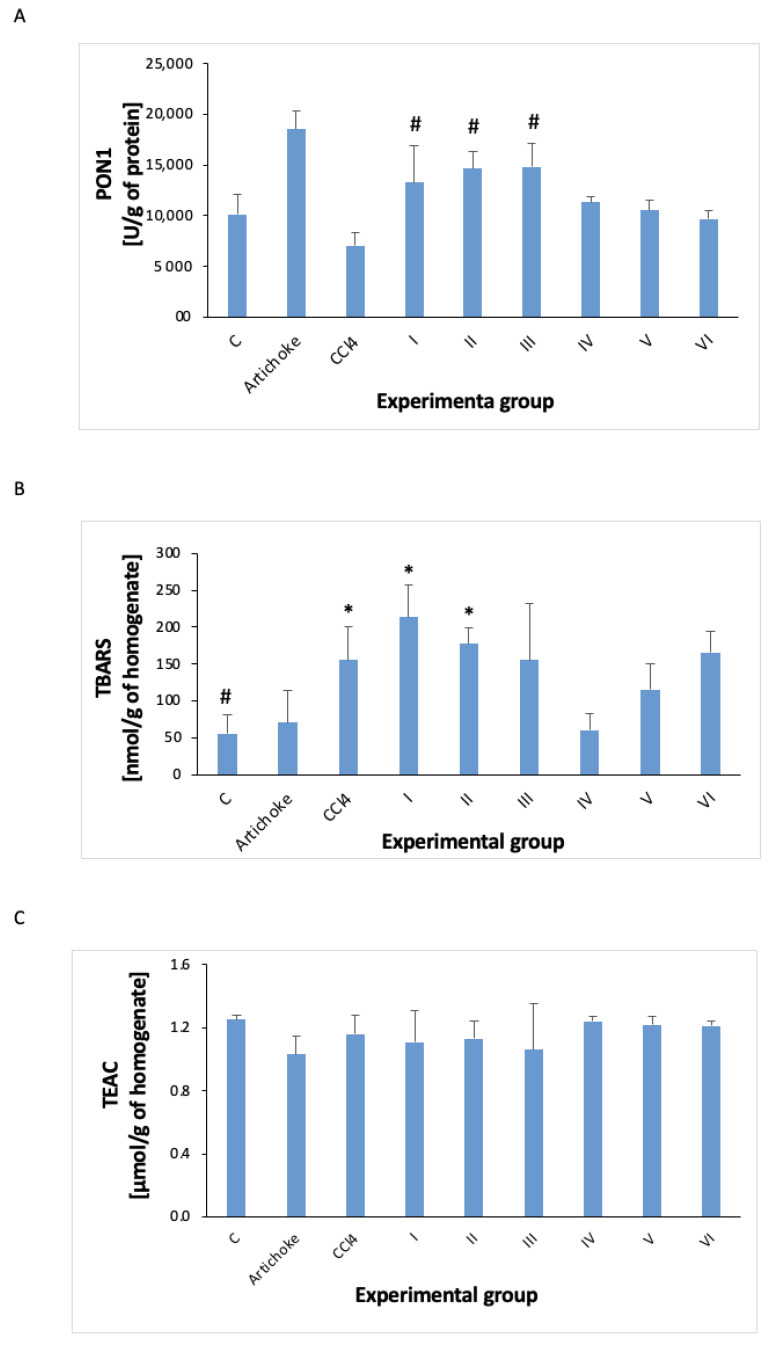
PON1 (**A**) activity and concentration of TBARS (**B**) and TEAC (**C**) in the liver homogenate in all experimental groups; * statistically significant difference in the studied groups versus control group; ^#^ statistically significant difference in the studied groups versus group after administration of carbon tetrachloride.

The following parameters of oxidative stress were investigated in this study of liver homogenate: protein; GSH; GST; TEAC; TBARS; SOD; catalase; GPx; PON1 (Figure 7, Figure 8 and Figure 9). 

In the case of glutathione-S-transferase activity (Figure 7), the carbon tetrachloride causes a slight reduction, and the earlier administration of the artichoke preparation significantly increased GST activity in groups I and II, and in group III, the increase was not statistically significant. Administration of the artichoke preparation after previous exposure to CCl_4_ caused only a slight effect on the activity of glutathione S-transferase (not statistically significant). 

CCl_4_ significantly reduces the SOD activity (Figure 7). The administration of artichoke before CCL_4_ at a dose of 0.5 g/kg bw resulted in a statistically significant increase, but in groups II and III, these changes were not statistically significant. Administration of the artichoke preparation for regenerative purposes caused statistically significant increases in SOD activity, equivalent to the activity observed in the control group. 

In the liver homogenate, TP, GSH, RSNO, -SH groups, TEAC and TBARS were determined. 

Total protein slightly decreases after the administration of CCl_4_, and the earlier administration of the artichoke preparation does not show a statistically significant effect on the level of TP. In the regenerative model, administration of the artichoke preparation brings the TP level closer to that observed in the control group (Figure 8). 

The administration of CCl_4_ slightly reduces the level of GSH after the administration of the artichoke preparation, but these changes are not statistically significant, as the changes in GSH were observed after the use of the higher artichoke preparation for regeneration purposes. 

When assessing the level of nitrosylated protein as a biomarker of oxidative stress, its level was not altered in liver homogenate by carbon tetrachloride or by artichoke preparation. 

The effect of CCl_4_ on the -SH level is not statistically significant in performed studies. 

The TEAC value in all studied groups did not show statistically significant differences. 

After administration of CCL_4_, a decrease in catalase activity was observed. In the artichoke preventive and regenerative activity model, the increase in the CAT activity due to large standard deviations was not statistically significant (Figure 7). 

CCl_4_ reduced the activity of paraoxonase 1 (Figure 9), but this change was not statistically significant. However, after the administration of the artichoke preparation before the administration of CCl_4_, its activity significantly increased. The administration of the artichoke preparation after the previous administration of carbon tetrachloride also increased the activity of this enzyme (statistically not significantly), bringing it to the level observed in the control group (changes are also statistically insignificant). 

The level of TBARS increased with the administration of CCl_4_. The administration of the previous artichoke preparation did not change it. The administration of the artichoke preparation for regenerative purposes caused a return to the control values, but due to the large standard deviation of the results, these changes were not statistically significant (Figure 9).

## 4. Discussion 

This study aimed to evaluate the protective and regenerative influence of commercially available artichoke leaf extract (*Cynara scolymus* L.) on the activity of selected antioxidants in plasma and liver homogenate. 

Oxidative stress contributes to the uncontrolled growth of reactive oxygen (higher levels of ROS) and nitrogen species production, and they can lead to the majority of damage to proteins, lipids, and nucleic acid structures [37,38]. As a consequence, there is observed a decrease in the body’s resistance, organ damage, and disease development [2,39,40]. Among other things, oxidative stress plays an important role in the pathogenesis of diseases of the liver, kidneys, and many other tissues [41,42]. Numerous studies have shown that the dose-dependency of CCl_4_ induces oxidative stress, degeneration, and liver necrosis [42,43]. It activates tumor necrosis factor (TNF-α), nitric oxide (NO), and transforming growth factors (TGF-α and -β) in cells [44]. The above-mentioned oxidation processes can be interrupted by antioxidants [45]. The liver is one of the most important organs in the human body and plays a vital role in maintaining and regulating homeostasis. Some herbal products have shown protective and regenerative effects against the hepatotoxicity caused by xenobiotics. Several herbal extracts have been suggested to offer protection against drug-induced liver damage. Among them, artichoke preparations have been found to protect hepatocytes from the effects of various toxins, such as carbon tetrachloride, which is a model compound known to damage parenchymal organs. Artichoke leaf extract (*Cynara scolymus*) is a popular herbal remedy that supports liver function in traditional folk medicine, and its hepatoprotective and antioxidant effects have been previously demonstrated through experimental and clinical studies. 

In this study, rats were treated with artichoke leaf extract orally for two weeks, which significantly reduced plasma transaminase enzyme activity induced after CCl_4_ treatment. The present study investigated the potential protective and regenerative effect of an artichoke preparation prepared from various parts of the plant on carbon tetrachloride-induced hepatotoxicity [46]. Carbon tetrachloride is a widely recognized and commonly used xenobiotic [42,47,48]. 

ALT is a cytosolic enzyme that is distributed in hepatocytes, while AST is localized in the mitochondria and cytoplasm of liver cells; thus, increased serum activities of these enzymes indicated that hepatocyte damage had reached the level of cell membranes and organelles [49]. The results of CCL_4_ administration are an increase in liver enzyme activities in the blood, loss of cell membrane permeabilization in the liver, and cellular leakage [50]. 

The increase in these enzymes to the value 400–4000 U/L may be observed as a result of chronic (chronic viral hepatitis, alcohol-related liver disease, autoimmune hepatitis, non-alcoholic fatty liver diseases, drugs, and toxins, non-steroidal anti-inflammatory drugs, antibiotics, and antifungals, HMG Co-A-reductase inhibitors, antiepileptic drugs antituberculous drugs, herbal medications, illicit drug use, Wilson’s disease, hemochromatosis, α1-antitrypsin deficiency, coeliac disease, glycogen storage disease, extrahepatic, hyperthyroidism, hypothyroidism, macro-aspartate aminotransferase, myopathy, strenuous exercise, hemolysis) and acute (acute viral hepatitis, ischaemic hepatitis, drug, and toxin-induced, acute Budd–Chiari syndrome, autoimmune hepatitis, fulminant Wilson’s disease, and liver diseases [51]. 

In our study, we have noted around a 10-time increase in ALT and around 8-time increase in AST levels in animals (control and positive control) as a result of exposure to the CCl_4_ (Figure 2A,B). Increased levels of AST and ALT in the serum are indicators of hepatic damage [52]. The administration of the artichoke leaf extract before the administration of CCl_4_ significantly reduced this effect. There was a significantly lower activity of these enzymes in the plasma of animals exposed to CCL_4_ and received extract after exposure (group IV–VI Figure 2A,B) compared to animals exposed to CCl_4_. 

The results from assays conducted for the experiment showed that intraperitoneal administration of CCl_4_ increases AST 10-fold and ALT 8-fold (due to the high variability of the results, these differences were not statistically significant). The administration of the artichoke preparation before the administration of CCl_4_ resulted in a reduction in the activity of these enzymes, but the reduction was not statistically significant. In Chowdhury et al.’s studies, a significant increase in the activity of these enzymes was also observed, but it was lower than ours, probably due to administering a lower dose of CCl_4_ [53]. The regenerative effect of the artichoke preparation (administration after the previous administration of CCl_4_) resulted in the return of ALT and AST activity to the control values. 

Alkaline phosphatase is an enzyme that is in a wide variety of tissues, including bone, liver, intestines, and kidney membrane-bound glycoprotein, that catalyzes the hydrolysis of phosphate monoesters [54]. In the case of hepatobiliary system diseases and disorders of the bile flow into the small intestine, alkaline phosphatase is of diagnostic significance. This enzyme is closely related to the lipid membrane [55]. It is involved in the metabolism of organic phosphates, with optimum action in the alkaline range. Elevated levels of ALP may indicate liver disease, bile secretion disorders, diseases of the skeletal system, and disseminated neoplasm (with bone metastases). The increase in alkaline phosphatase activity induced by the administration of CCl_4_ (Figure 3) was significantly lower than that of ALT and AST and was less than 1.5 times. Previous research has shown similar results, and the effect of the artichoke preparation on the activity of this enzyme was unclear due to the wide dispersion of the results obtained. 

In addition, elevated serum ALP levels associated with liver and biliary damage have been observed, while serum creatinine and blood urea nitrogen levels were particularly used to identify kidney injury [56]. Another enzyme activity used to evaluate the antioxidant activity of the artichoke preparation was SOD. It is known that all aerobic organisms contain superoxide dismutase. SOD is widely distributed in both plants and animals, and in animals, it is found in many tissues, including the brain, liver, heart, red blood cells, pancreas, intestines, muscles, and kidneys. SOD serves as the first line of defense against reactive oxygen species; these proteins catalyze the dismutation of superoxide anion free radicals into molecular oxygen and hydrogen peroxide. They are also used to treat oxidative stress due to their smaller size and longer half-life. Several studies have been carried out to reveal the therapeutic potential and physiological importance of SOD. Current research reveals potential therapeutic applications of SOD in the prevention and control of various diseases, for example, by increasing the level of antioxidants in plant products through genetic modifications [57]. 

In our studies, the activity of SOD decreased by 41% after administration of CCl_4_ in other studies, and the decrease in other units was about 60%. The administration of the artichoke preparation both before and after CCl_4_ increased the activity of this enzyme to the values observed in the control group, although the administration of this preparation for regenerative purposes was not statistically significant due to the large difference in individual measurements. 

GSH is a substance used at the cellular level and occupies a primary position in the body’s defense systems. It is mainly concentrated in the liver, where it plays a key role in detoxification functions. GSH is a tripeptide that protects hepatocytes against the harmful effects of free radicals, toxic substances, and peroxides. It has been shown that GSH plays a role in the detoxification of xenobiotics, such as ethanol and tobacco smoke [28,58,59]. Low levels of GSH in the blood indicate disease and imply that the body cannot effectively neutralize free radicals. Decreased levels of plasma GSH are observed in patients with liver cirrhosis [60,61]. 

In the conducted studies, CCl_4_ caused a 39% decrease in glutathione levels. Only the highest dose of the artichoke preparation (1.5 g/kg b.w) was able to restore the glutathione level to normal, while no increase in glutathione recovery was observed after the previous administration of CCl_4_. In the studies by Mehmetcik et al., the extract was not found to affect cellular GSH but reduced GSH loss. Artichoke extract has been found to increase glutathione peroxidase (GSH-Px). In the case of prior use of CCl_4_, it was observed that the GSH content also increased enzyme activity. This indicates that the hepatoprotective effect of artichoke leaf extract is not only associated with the induction of GSH-Px but also with direct antioxidant properties [46]. Mehmetcik et al. observed in their experiment, in which the animals were administered the same amount of CCl_4_, that the hepatic GSH level was higher in the case where rats received previous artichoke leaf extract in an amount of 1.5 g/kg bw/day [46]. Similarly, in liver homogenates of rats exposed to toxic diethyl-nitrozoamine, GSH levels were significantly higher when animals were treated with bloom head or leaf extract from artichoke, which also indicated the protective effect of this plant [62]. 

Glutathione S-transferases are the main phase II detoxification enzymes mainly found in the cytosol. In addition to their role in catalyzing the conjugation of electrophilic substrates to glutathione, these enzymes also have several other functions that influence their activity. Engy et al. investigated the effect of paracetamol on GSH and GST, and they observed a decrease in these parameters. However, a direct comparison with our results is not possible due to the use of different xenobiotics causing oxidative stress and other units for presenting the results. After the administration of CCl_4_, the activity of GST decreased significantly, but after the application of the artichoke preparation, it returned to the control value in all the studied groups [63]. 

Physiological concentrations of nitric oxide and temporary stimulation of its production play a positive role in cell homeostasis. However, too intense production can contribute to the formation of degenerative processes and tissue damage caused by the peroxynitrite anion (formed in the reaction of nitric oxide with superoxide in inflammatory processes) [60]. 

The action of NO in the case of liver disease is not entirely clear. During acute liver failure, NO also reaches the systemic circulation due to the release of substances such as NO. Some studies have shown that NO reduces rat liver injury induced by ethanol [64]. Other studies suggest that increased NO levels are observed in patients with advanced cirrhosis state [65]. Administration of CCl_4_ in the Bakdemir and Çetin studies increased the NO level by approximately 70% [66]. Assessment of the influence of CCl_4_ on the level of NO showed its approximately two-fold increase. The regenerative administration of the artichoke preparation resulted in a statistically significant reduction in NO compared to that after the administration of CCl_4_. On the other hand, administration of the artichoke preparation after the earlier administration of CCl_4_ resulted in maintaining a high level of NO, but it was not statistically different from the control values. 

As it turns out, NO levels and SOD activity are associated with each other [67]. During inflammation, NO is produced in increased amounts and may cause tissue injury by reacting with superoxide to yield peroxynitrite, a powerful toxin. Meanwhile, superoxide dismutase scavenges superoxide and inhibits the formation of peroxynitrite. 

Currently, there are no reports about the effect of an artichoke extract on plasma NO levels. In the conducted studies, no statistically significant changes in the level of nitrosylated protein were observed. 

The thiobarbituric acid reacting substances test is widely used as a general measure of lipid peroxidation in biological fluids and is assessed as a measure of oxidative damage to red blood cells. TBARS is perhaps the oldest and one of the most widely used tests to measure the end product of lipid peroxidation, malondialdehyde (MDA), a reactive aldehyde produced by the lipid peroxidation of polyunsaturated fatty acids. The TBARS measurement methodology has been criticized for its low sensitivity and selectivity, as several non-MDA species from biological samples may react with TBA, and some artifacts of MDA production during the test have also been raised. When investigating beetroot juice as an antioxidative stress agent, TBARS was reduced by 38% in rats that were exposed to CCl_4_ after pretreatment with beetroot juice before dosing. The rats pretreated with juice and treated with N- Nitrosodiethylamine (a compound that induces oxidative stress) had a further increase in TBARS [68]. In our study, we observed an increase in TBARS after the administration of CCl_4_, but this value did not reach statistical significance compared to the other values obtained in the studies. 

In the conducted studies, administration of CCl_4_ caused a slight decrease in TEAC (about 10%). Betancor-Fernandez et al. found good coefficients of the linear regression between the TEAC values and the total phenolics in test formulations [69]. On the other hand, in our study, the artichoke preparation administered before CCl_4_ increased the TEAC value statistically significantly about the control value and after the application of CCl_4_. Our research also included the evaluation of the effect of artichoke preparation in the protection and regeneration of the liver resulting from exposure to carbon tetrachloride assessed in the liver homogenate, an organ that was damaged by this solvent (histological studies not included in the present study). The results of the influence of the artichoke preparation on the markers of the activity of CCl_4_ based on liver homogenate tests have been carried out in rats only in a few studies. This causes problems in comparing lower results with the results of other authors. 

At the beginning of the sentence, the homogenate was marked with the protein level in it. No statistically significant effect of CCl_4_ on the protein level was observed. 

In all studied groups, the activity of catalase differed in a statistically insignificant manner, even though after administration of CCl_4_, the activity of this enzyme decreased two times. After the administration of the artichoke preparation, the catalase activity in all study groups was not significantly different from that in the control group. 

Research on the protective effect against oxidative stress using liver homogenate and rosemary essential oil (artichoke has not been tested in such a system) found that catalase activity was reduced after the administration of CCl_4_, but contrary to our study, it was statistically significant [70]. 

Endogenous antioxidants such as GSH play an important role in counteracting CCl_4_. In these studies, silimarol did not alter GSH levels [71]. In our study, neither CCl_4_ influenced the level of GSH in the liver homogenate nor did the artichoke preparation statistically significantly change this parameter. 

In the studies of Al-Sayed et al., similarly to ours, slightly decreased GPX activity was observed, and in our studies, the artichoke preparation had a statistically insignificant effect on this parameter [72]. MDA is an intermediate product of lipid peroxidation and can impair cell composition, structure, and function. In this study, the administration of CCl_4_ reduced MDA levels by about two-fold [73]. The situation in our experiment was similar. On the other hand, the artichoke preparation administered before CCl_4_ increased the TBARS levels, although not statistically significant, while the administration of the artichoke preparation after carbon tetrachloride increased the level of TBARS proportionally to the dose of the preparation. 

We did not find information on the effect of CCl_4_ on TEAC levels in rat liver homogenate in the available literature. In our study, we did not show any changes in the TEAC test results. 

In the current literature, publications on the effects of CCl_4_ on PON1 levels, -SH groups, and nitrated protein in rodent liver homogenate were not available. 

In the conducted studies, the results of PON1 after the administration of CCl_4_ showed a decrease in blood pressure, but the differences compared to the control group were not statistically significant. However, the administration of the artichoke preparation before CCl_4_ significantly increased the PON1 values. As for the levels of nitrosylated proteins and the -SH groups, no interesting results were observed. 

In conclusion, the studied preparation of *Cynara scolymus*, due to its high antioxidative potential, has liver protective and regenerative properties, which were confirmed by biochemical analysis of ALT, AST, GSH, GST SOD, and TBARS, particularly in the plasma of rats. The artichoke extract also exhibited liver regenerative properties, as indicated by lower levels of liver enzymes and SOD activity in animals with damaged liver due to exposure to the toxic effects of CCl_4_ and then treated with the extract, compared to those which did not receive it. 

The diversity among oxidative stress parameters presented in this study was likely observed because the mechanisms sensitive to oxidative stress act comprehensively. In case of shortage or low activity of one antioxidant enzyme, the other mechanisms come into play to stimulate and remove excess free radicals through the appropriate pathways. 

Despite significant progress in pharmacotherapy using synthetic drugs, phytotherapy still holds an undeniable place in the treatment of many diseases. In addition to their demonstrated medicinal properties, preparations from plants are used for protection during drug therapy, which may otherwise cause damage to parenchymal organs. 

The results showed that artichoke has antioxidative, hepatoprotective, and regenerative effects on liver cells. Recent in vivo studies on the toxicity of artichoke (*Cynara scolymus* L.) showed no evidence of genotoxicity and mutagenicity in cells of mice when aqueous leaf extracts were given in a dose below 2 g/kg bw [74]. 

Commercial products containing artichoke leaf extract may have different contents of active compounds and, thus, different antioxidant potentials. Speroni et al. have demonstrated that the antioxidant potential of commercially available preparations was greater when the contents of the chlorogenic and caffeoylquinic acids were higher [75]. Therefore, other extracts with higher contents of these compounds may be more beneficial in inhibiting the harmful effects of oxidative stress. 

Because synthetic drugs used today often cause hepatotoxicity, concomitant administration of *Cynara scolymus* extracts as a source of substances that inhibit this undesirable effect can reduce the damage while maintaining adequate quantities and increase the therapeutic relevance of these drugs. 

## Figures and Tables

**Table 1:** HPLC gradient used for the determination of chlorogenic acid.

Time (min)	Phase A (%)	Phase B (%)
0	93	7
18	93	7
22	75	25
33	75	25
35	0	100

## Data Availability

The data presented in this study are available in this article.
